# Does Intraspecific Size Variation in a Predator Affect Its Diet Diversity and Top-Down Control of Prey?

**DOI:** 10.1371/journal.pone.0020782

**Published:** 2011-06-08

**Authors:** Travis Ingram, William E. Stutz, Daniel I. Bolnick

**Affiliations:** 1 Department of Zoology and Biodiversity Research Centre, University of British Columbia, Vancouver, British Columbia, Canada; 2 Section of Integrative Biology, University of Texas at Austin, Austin, Texas, United States of America; 3 Howard Hughes Medical Institute, Section of Integrative Biology, University of Texas at Austin, Austin, Texas, United States of America; University of Western Ontario, Canada

## Abstract

It has long been known that intraspecific variation impacts evolutionary processes, but only recently have its potential ecological effects received much attention. Theoretical models predict that genetic or phenotypic variance within species can alter interspecific interactions, and experiments have shown that genotypic diversity in clonal species can impact a wide range of ecological processes. To extend these studies to quantitative trait variation within populations, we experimentally manipulated the variance in body size of threespine stickleback in enclosures in a natural lake environment. We found that body size of stickleback in the lake is correlated with prey size and (to a lesser extent) composition, and that stickleback can exert top-down control on their benthic prey in enclosures. However, a six-fold contrast in body size variance had no effect on the degree of diet variation among individuals, or on the abundance or composition of benthic or pelagic prey. Interestingly, post-hoc analyses revealed suggestive correlations between the degree of *diet* variation and the strength of top-down control by stickleback. Our negative results indicate that, unless the correlation between morphology and diet is very strong, ecological variation among individuals may be largely decoupled from morphological variance. Consequently we should be cautious in our interpretation both of theoretical models that assume perfect correlations between morphology and diet, and of empirical studies that use morphological variation as a proxy for resource use diversity.

## Introduction

A fundamental goal in evolutionary biology is to understand the origin, maintenance, and consequences of variability within populations. The key forces shaping genetic and phenotypic variation within and between populations have been identified, as have many of the consequences of this variation for adaptation and speciation [1], [2]. In contrast, we are only beginning to learn how intraspecific variation affects ecological interactions and processes [3], [4]. Genetic polymorphism and heterogeneous environmental conditions can, singly or in concert, produce variation in phenotypic traits involved in interspecific interactions. Variation in such traits implies that conspecific individuals experience different interactions, such as relative reliance on alternate prey species [5], or vulnerability to different predators [6] or parasites [7]. A major task facing ecologists is to determine how this intraspecific variation affects the structure and dynamics of populations, communities, and ecosystems.

A small but growing set of theoretical models suggests that intraspecific variation can have profound effects on population [8]–[10], predator-prey [9], [11]–[13], and host-parasite dynamics [8]. Genetic variation permits trait evolution, which can alter the mean strength of interspecific interactions or allow coevolutionary dynamics that may promote coexistence [11]. Trait variance per se can also affect population size, stability or interspecific interactions when relationships between traits and interaction strengths are non-linear [4], [12]. Finally, diversity within populations may have effects analogous to effects of species richness on ecological dynamics, either via niche complementarity or sampling of distinctive phenotypes [14], [15]. Theoretical models that investigate the effects of trait variation typically assume either a 1∶1 relationship between individuals' phenotypes and their ecological interactions, or that the phenotype itself is a direct measurement of the interaction (e.g. [9], [11]). At present it is not clear how more realistic weak-to-moderate correlations affect the ecological impact of phenotypic variation.

Experimental manipulations of intraspecific genotypic diversity have supported many of these theoretical predictions. These studies have demonstrated that genotypic diversity can enhance population productivity [16] or stability [17], [18], increase the abundance or diversity of higher trophic levels [16], alter rates of nutrient cycling [19], and allow eco-evolutionary feedbacks affecting predator-prey dynamics [20]. The majority of studies examining effects of intraspecific variation on trophic interactions have focused on the bottom-up effects of trait variation in resource species (e.g. [16]). However, manipulations of predator species richness suggest that diversity can either enhance or reduce the strength of top-down control [21]–[23], possibly depending on the degree of omnivory in the predators.

Most experimental manipulations have used clonal genetic diversity in asexual species as a measure of intraspecific variation (but see [18], [24]). Comparatively few studies have directly tested for ecological effects of intraspecific *phenotypic* variation, despite its central role in ecological theory pertaining to intraspecific variation [25]. The few studies to focus on phenotype have compared effects of morphologically divergent populations [23], [26], [27] rather than the degree of variation within a natural population. Manipulations of clonal lineages or divergent populations typically affect multiple covarying phenotypic characters, whereas by manipulating phenotypic variance within a population we can largely isolate the effects of variance in a single ecologically relevant trait.

The threespine stickleback (*Gasterosteus aculeatus*) has undergone recent and dramatic evolutionary divergence among north temperate marine, stream, and lake habitats [28]. Sympatric pairs of benthic and limnetic specialist species have evolved in several lakes in British Columbia [29], and individuals within generalist populations vary extensively in their use of benthic vs. limnetic prey [30], [31]. This diet variation is related to intraspecific variation in morphological traits including body size, body shape, gape width and gill raker length and number [30]–[33]. For example, stomach content and stable isotope analyses show that larger fish tend to feed on more benthic prey and at a higher trophic position [30], [31].

Levels of morphological and ecological variance differ among stickleback populations. Trophic traits (gape width and gill raker morphology) tend to be more variable in lakes predicted to have a greater diversity of habitats and prey types [34], [35], and lake populations on Vancouver Island differ significantly in measures of within-population diet variation (L.K. Snowberg and D.I. Bolnick, unpublished data). Because trait and diet variance differ among populations [23], [36], [37], it is biologically reasonable to ask whether intraspecific trait variance alters the impact of stickleback on their prey community. Here, we describe a field experiment testing for short-term ecological effects of body size variance within an age cohort of threespine stickleback in enclosures. We show that stickleback exert top-down control on benthic prey and that individual fish size is correlated with prey size. Surprisingly, we did not detect any effect of a body size variance manipulation on diet variation or on the prey community.

## Materials and Methods

### Ethics statement

Animal use protocols in this experiment were approved by the University of Texas Institutional Animal Care and Use Committee (permit # 07100201).

### Experimental design and sample collection

In late May, 2009, we established experimental enclosures in Blackwater Lake, on Vancouver Island, British Columbia (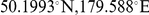). Typical of most coastal lakes, Blackwater Lake contains a single generalist population of stickleback that is morphologically intermediate between benthic and limnetic species. Eighteen 3.3
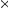
3.3 m square enclosures were constructed with 1/16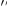 seine netting following [37] and [38]. Each enclosure was sealed to the benthic mud at a depth of 1–1.5 m, allowing fish access to both benthic and open-water microhabitats. Enclosures were arranged in six spatial blocks along 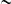
300 m of shoreline, with the three enclosures within each block one to two meters apart. Any accidentally enclosed fish were removed with minnow traps and dipnets. Two enclosures per block were randomly selected to receive high- and low-variance stickleback populations, while the third, ‘fishless’ enclosure was left free of stickleback to allow detection of overall top-down control of prey.

**Figure 1:**
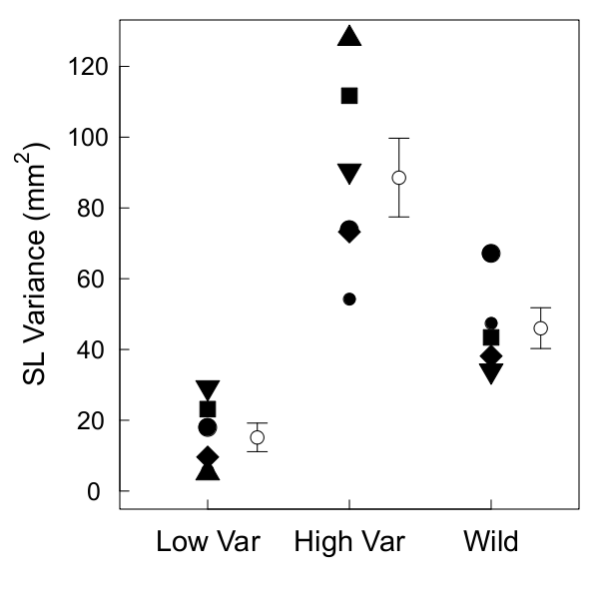
Variance in standard length of threespine stickleback in the high (HV) and low size variance (LV) enclosures. For comparison, we show variances for samples of wild-caught fish from outside the five of the six blocks. Symbols represent spatial blocks and can be compared with Figs. 2, 4 and 5, and error bars indicate 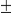 1 SE.

Before stocking the enclosures with fish, we delineated three size categories based on the 25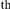 and 75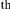 percentile of the distribution of standard lengths (SL) of 81 stickleback measured the day prior to stocking. We then used minnow traps to collect 360 experimental fish, almost all of which were likely one-year old adults (D.I. Bolnick, unpub. data). We sorted these fish into small (29–49 mm SL), medium (49–59.5 mm) and large (59.5–67 mm) size classes. We stocked high-variance (HV) enclosures with a mixture of 15 large fish and 15 small fish, and low-variance (LV) enclosures with 30 medium-sized fish. 30 fish per enclosure approximates natural densities in Blackwater Lake, and leads to growth rates and prey consumption similar to outside the enclosures [37]. This protocol produced a strong contrast in final SL variance between HV (mean variance = 88.6 mm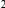) and LV (15.2 mm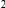) enclosures (Fig. 1; 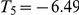, 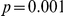). There was also a small, unintended difference in mean SL (HV: 49.7 mm; LV: 52.0 mm; 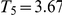, 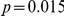) but no difference in total stickleback biomass (HV: 35.8 g; LV: 34.1 g; 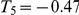, 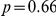). After adding the fish we left the enclosures undisturbed for 21 days.

We removed all fish from the enclosures with minnow traps and dipnets, checking traps at least every 3 hours to ensure that stomach contents accurately reflected pre-capture foraging [37]. Recapture rates were high (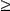
25 of 30 fish, except for one enclosure with 19 recaptures), and did not differ between HV and LV enclosures (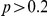). Some LV enclosures contained one or two fish that were too small to have been stocked. We included these fish in all analyses because they represented a real component of the enclosed stickleback populations, but their exclusion did not qualitatively alter any results. We simultaneously collected a total of 75 ‘wild-caught’ stickleback using minnow traps placed outside each block of enclosures. Fish were euthanized with an overdose of buffered MS-222, then frozen for later processing.

The day before fish collection, we sampled zooplankton with morning and evening horizontal tows of a plankton net 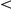
1 m below the surface (each 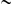
100 L). The two samples were aggregated for analysis, but separate analyses of morning and evening zooplankton yielded similar results. We sampled benthic prey immediately after fish removal, using a small aquarium net to scoop 450 cm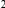 of benthic mud to a depth of 2 cm. We diluted each mud sample to a constant volume (2.5 L), mixed it thoroughly and subsampled 20% of this volume. Within 24 hours, each sample was examined in dissection trays for a total of 45 person-minutes, sufficient to pick essentially all benthic organisms. Zooplankton and benthic invertebrate samples were taken from inside each enclosure and immediately outside each block, and were frozen for later identification and enumeration.

### Community analyses: benthic invertebrates and zooplankton

We examined benthic invertebrate and zooplankton samples under a dissecting microscope, identifying and enumerating all potential diet items to the lowest feasible taxonomic level. We also estimated the distribution of body sizes in the prey communities. We measured lengths of individual prey of taxa that varied considerably in size (roughly, 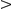
1 mm standard deviation of individual prey lengths), and substituted an average length (based on 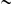
20 individual measurements) for less variable prey types. Prey size was quantified as log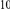-transformed mass, estimated using published taxon-specific length-weight regressions [39], [40].

For each benthic invertebrate and zooplankton sample we quantified total density (no. sample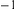), mean size, and variance in size. We also calculated Shannon diversity and its components, species richness (log[number of species]) and evenness (diversity divided by richness). For each of these variables, we used a linear mixed-effects model with spatial block as a random factor and experimental treatment as a fixed effect. We had four treatments (HV and LV, fishless, and outside enclosures), but we restricted analyses to three planned orthogonal contrasts. First, we tested for an enclosure effect by contrasting the outside sample with the mean of the three enclosed samples in a block. Second, we tested for a stickleback effect on the invertebrate community (top-down control) by contrasting the fishless treatment with the mean of the HV and LV treatments. Finally, we tested for an effect of size variance by contrasting the HV and LV treatments. Response variables were transformed where appropriate, normality of residuals was evaluated using quantile plots, and homogeneity of variances among treatments was confirmed with Bartlett's Test. All statistical analyses were conducted in the R environment [41].

We also used redundancy analysis (RDA, implemented in the R ‘vegan’ package) to test for effects of our treatments on benthic invertebrate and zooplankton community composition [42]. We used our experimental treatments as conditioning variables to predict each community matrix composed of relative frequencies of each taxon. In order to test for compositional effects of our three planned contrasts, we refit the RDA output as a multiple response linear model, and used MANOVA to test the significance of each contrast.

### Diet analyses: Does individual stickleback morphology affect individual diets?

We thawed each stickleback and measured standard length and body depth to 0.1 mm. We also counted gill rakers on the first gill arch, and calculated the average length of the three longest gill rakers, measured to 0.025 mm under an ocular micrometer. We then calculated residuals from linear regressions of body depth, gill raker number and log-transformed gill raker length against SL, yielding three size-corrected traits (hereafter ‘body depth’, ‘gill raker number’ and ‘gill raker length’). Means and variances of these traits did not differ significantly among size variance treatments (all 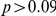).

Prey items in the stomach contents of each stickleback were counted and identified to the same taxonomic categories as the benthic invertebrate and zooplankton samples (35 categories in total). We also measured each prey item if possible, and substituted taxon-specific average lengths for the 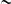
50% of prey items too damaged to measure. We used the same protocols described above to estimate the prey size distribution and mean prey size (in log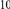 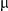
g) of each fish.

To determine the strength of the diet-morphology relationship, we calculated the correlation between individual fish size and mean prey size among the 75 wild-caught fish. We also conducted a linear regression of prey size against fish size in each enclosure population, and tested whether this slope differed between HV and LV treatments. For analyses of diet variables, we used a linear mixed-effect model with spatial block as a random effect and size variance treatment as a fixed-effect.

As diet variation may involve dimensions other than prey size, we evaluated the relationship between prey taxonomic composition and morphology by computing the proportional dissimilarity PDS (the complement of the proportional similarity, PS) between the diets of all pairs of individuals 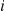 and 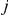:
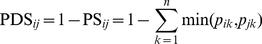
(1)where 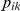 and 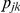 are the proportions of prey type 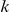 (of 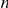 total prey types) in the diets of each individual [30], [43]. PDS ranges from 0 (complete diet overlap) to 1 (no shared diet items). We calculated the matrix correlation between PDS and the absolute size difference between individuals 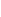
SL
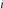
−SL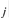, and tested for significance using a Mantel test with 9,999 permutations. We repeated both size- and taxon-based diet analyses using body depth, gill raker number and gill raker length, to compare body size to size-independent trophic morphology as a predictor of diet.

### Diet analyses: Does stickleback size variance affect diet diversity?

We expected any effects of increased stickleback size variance on prey density and composition to be mediated by increases in the population niche width and/or degree of individual specialization (diet variation). We tested whether size variance did in fact affect measures of diet variation based on both prey size and taxonomic composition. For prey size data, we first calculated the population's total niche width (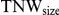) as the variance in size of all prey consumed by the population. We then decomposed 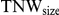 into its within- and between-individual variance components (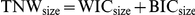), where 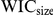 is the average variance of prey sizes used by an individual and 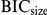 is the variance among individuals' mean prey sizes, both weighted by the number of prey items consumed. We then calculated the degree of diet variation as 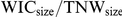, where a value of 1 indicates no individual specialization and values approach 0 as individuals sample a narrower range of 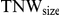 [44], [45].

We calculated the analogous indices (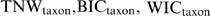 and 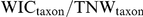) for the categorical diet data, using the Shannon diversity index as a proxy for variance (for details see [45]). We calculated the index E as an alternative measure of diet variation with better-known statistical properties and a more intuitive interpretation [33]. E is the mean proportional dissimilarity in diet (PDS from Eqn. 1) between all pairs of individuals, and ranges from 0 (no diet variation) to 1 (high diet variation). We tested whether stickleback size variance affected the degree of individual diet variation in prey size (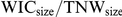 and 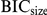) or prey taxa (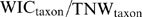 and E), or the population niche width (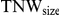 or 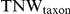).

## Results

### Community analyses: benthic invertebrates and zooplankton

**Figure 2:**
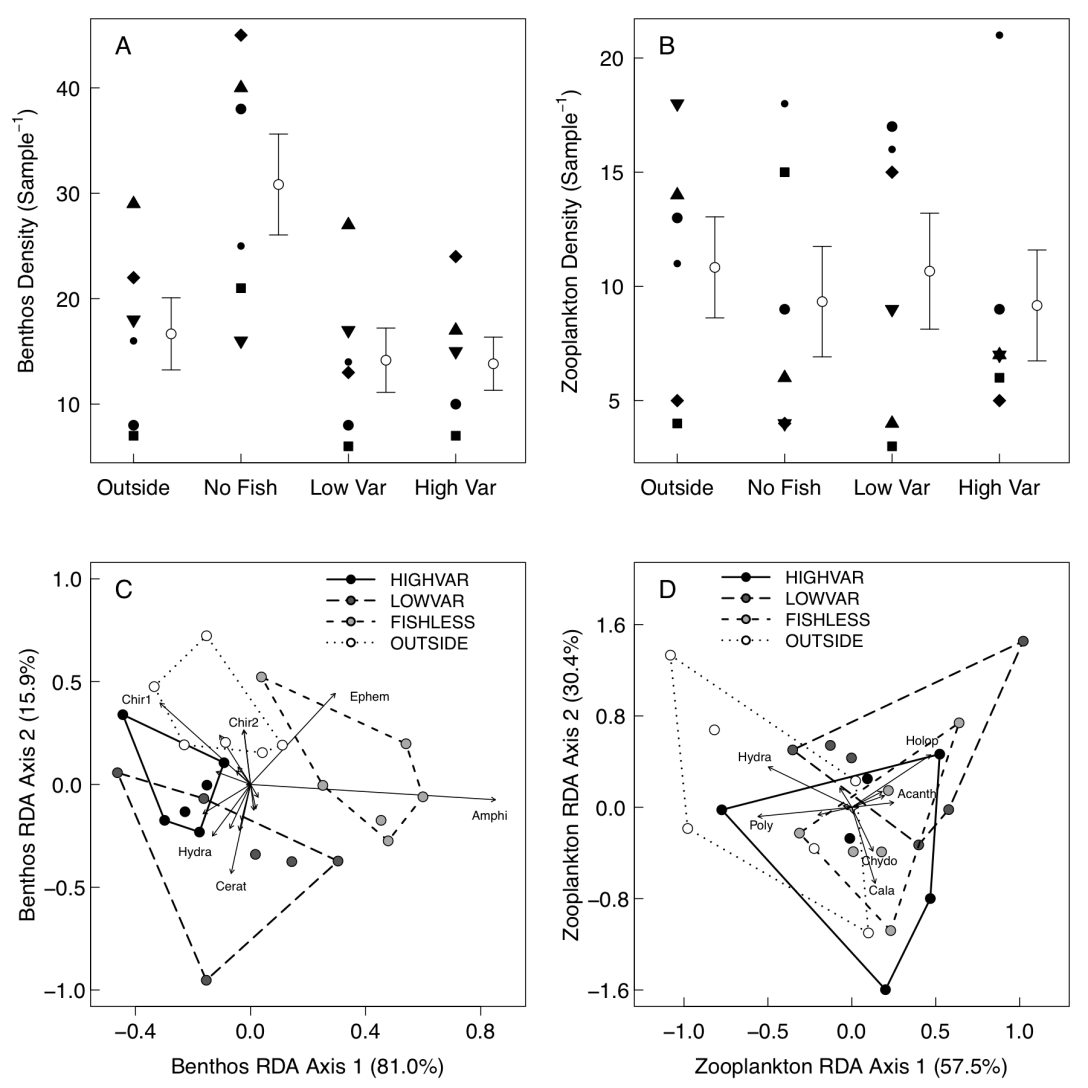
Effects of enclosures, stickleback presence and stickleback size variance on the prey community. Shown are densities of a) benthic invertebrates and b) zooplankton (Mean 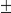 1 SE), and taxonomic composition of c) benthic invertebrates and d) zooplankton. The lower panels present the first two axes from redundancy analyses, showing the percentage of variance explained by each axis. Treatments are indicated both by shading of points and the line style of convex hulls. Arrows indicate the position of individual prey taxa in coordinate space; for clarity, only the six taxa farthest from the origin in each panel are labeled. Chir1 = chironomid larvae, subfamily chironominae; Chir2 = chironomid larvae, subfamily tanypodinae; Cerat = ceratopogonid larvae; Ephem = ephemeroptera larvae; Amphi = amphipod; Hydra = hydracarinid mite; Cala = calanoid copepod; Holop = *Holopedium gibberosum*; Acanth = *Acantholebris* sp.; Chydo = *Chydoris* sp.; Poly = *Polyphemus* sp.

There were no enclosure effects on benthic invertebrate abundance (Fig. 2A), size structure or diversity (planned contrasts of samples from inside vs. outside the enclosures). There was an enclosure effect on benthos composition (RDA; 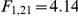, 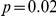; Fig. 2C), largely due to a lower relative abundance of larval chironomids inside enclosures. The zooplankton community had higher evenness (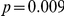), marginally greater size variation (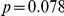) and slightly different composition (RDA; 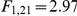, 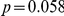; Fig. 2D) inside vs. outside enclosures, but did not differ in density (Fig. 2B), diversity, richness or mean size (all 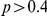). Thus, while the enclosures did affect some aspects of the prey community, stickleback experienced a reasonably natural foraging environment with access to multiple benthic and pelagic prey types.

**Figure 3:**
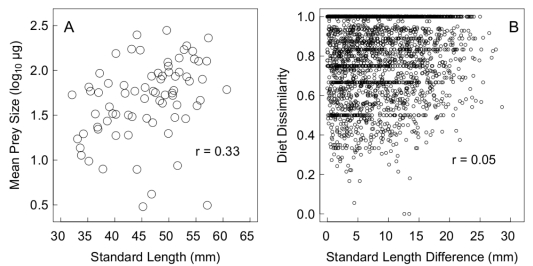
Relationships between size and diet in 75 wild-caught stickleback from Blackwater Lake. a) Mean prey size is positively correlated with standard length, while b) Pairwise diet dissimilarity between individual stickleback is positively but non-significantly correlated with difference in standard length.

We found evidence that stickleback exert top-down control on multiple aspects of their prey community. On average, enclosures with stickleback contained fewer than half as many benthic invertebrates (14 per sample) as fishless enclosures (30.8 per sample; 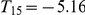, 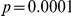; Fig. 2A). Fish presence had no effect on the mean or variance in benthos sizes (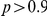) or on overall benthic invertebrate diversity (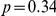). However, fish presence affected how benthic diversity was partitioned, increasing evenness (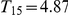, 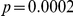) and weakly decreasing richness (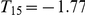, 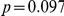). Fish presence also strongly impacted overall benthos composition (RDA; 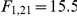, 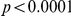; Fig. 2C), in particular by decreasing the relative abundance of common grazers such as amphipods and mayfly larvae. There were no significant effects of fish presence on zooplankton density (Fig. 2B), size structure, diversity or composition (Fig. 3B; all 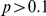). The lack of zooplankton effects may be an experimental artifact arising from the permeable net enclosures, though at higher densities stickleback can suppress zooplankton densities in similar enclosures [37].

Despite this evidence that stickleback presence affects benthic prey abundance and composition, our size variance manipulation had no effect on any aspect of the prey community. HV and LV treatments did not differ in benthic invertebrate (mean HV: 13.8; mean LV: 14.2; 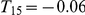, 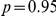; Fig. 2A) or zooplankton density (mean HV: 9.2; mean LV: 10.7; 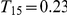, 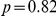; Fig. 2B), mean size, variance in size, or any aspect of diversity (all 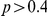). Redundancy analysis showed only a marginal effect of variance treatment on benthic invertebrate composition (RDA; 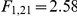, 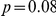; Fig. 2C) and no effect on zooplankton composition (RDA; 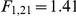, 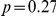; Fig. 2D). Mean stickleback stomach content mass did not differ between variance treatments (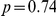), consistent with this lack of a size variance effect on the prey community. Detailed statistical results for community variables are presented in Table 1.

**Table 1:** Treatment effects on food web variables.

	HV – LV	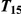	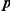	Fish – NF	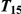	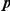	In – Out	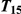	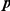
Benthos Density*	−0.011	−0.06	0.95	**−0.822**	**−5.16**	**0.0001**	0.122	0.82	0.43
Benthos Mean Size	0	0	1.00	−0.003	−0.03	0.98	−0.036	−0.32	0.75
Benthos Var Size	0.006	0.1	0.92	0.003	0.06	0.95	−0.02	−0.38	0.71
Benthos Diversity	0.031	0.23	0.82	0.118	0.98	0.34	−0.054	−0.48	0.64
Benthos Richness	0.005	0.04	0.97	*−0.17*	*−1.77*	*0.097*	0.039	0.43	0.68
Benthos Evenness	0.014	0.48	0.64	**0.126**	**4.87**	**0.0002**	−0.039	−1.6	0.13
Zooplankton Density*	0.077	0.23	0.82	0.068	0.24	0.82	−0.143	−0.53	0.60
Zooplankton Mean Size	0.032	0.44	0.66	0.057	0.9	0.38	−0.011	−0.19	0.85
Zooplankton Var Size	0.009	0.37	0.72	0.025	1.17	0.26	*−0.037*	*−1.89*	*0.078*
Zooplankton Diversity	−0.098	−0.47	0.64	−0.162	−0.9	0.38	0.134	0.79	0.44
Zooplankton Richness	−0.151	−0.65	0.52	−0.123	−0.62	0.55	0.044	0.23	0.82
Zooplankton Evenness	0.022	0.78	0.45	−0.04	−1.6	0.13	**0.07**	**2.98**	**0.009**

Results of linear mixed-effects modeling of food web variables as a function of experimental treatment. Effect sizes are given for the three planned contrasts: high vs. low SL variance (HV–LV); fish present vs. absent (Fish – NF); enclosed vs. outside enclosures (In – Out), while the random block effect was included in the model but is not shown. Significant or marginally significant effects are indicated in boldface (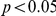) or italics (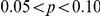). Variables marked with an asterisk (*) were log-transformed to improve normality.

### Diet analyses: Does individual stickleback morphology affect individual diet?

We confirmed that large stickleback tend to consume larger prey. In wild-caught fish, there was a significant positive correlation between SL and mean prey size (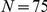, 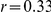, 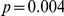; Fig. 3A). The correlation was slightly stronger (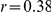) when fish were weighted by the number of items in their stomach to reduce the influence of several possible outliers (e.g. large fish with only a single small diet item). Within enclosures, the slope of the relationship between prey size and fish size tended to be positive (one-sample t-tests; mean HV: 0.017; 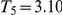, 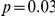; mean LV: 0.030; 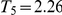, 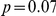), but did not differ between treatments (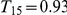, 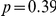). Mean prey size in wild-caught fish was not significantly related to gill raker number (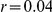, 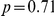), gill raker length (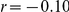, 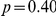), or body depth (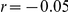, 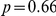).

We also confirmed that morphology affects the taxonomic composition of diets of wild-caught fish. However, this effect was weak for standard length, with a non-significant positive relationship between pairwise size differences between individuals and pairwise diet dissimilarity (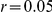; Mantel test, 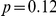; Fig. 3B). There were weak but significant positive relationships between diet dissimilarity and dissimilarity in gill raker number (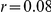, 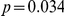) and length (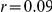, 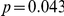) but not body depth (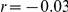, 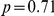). These correlations are comparable in strength to those found among 265 stickleback from another population in the watershed [30].

### Diet analyses: Does stickleback size variance affect diet diversity?

**Figure 4:**
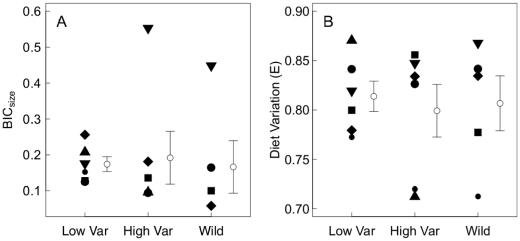
Effect of stickleback size variance on population-level diet variation. a) variance in mean prey size among individuals (BIC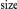) and b) mean pairwise diet dissimilarity based on prey frequency data (E). Symbols correspond to spatial blocks, and ÒwildÓ samples correspond to fish caught outside five of the six blocks. Mean 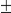 1 SE.

As individual stickleback size is correlated with prey size (and weakly with taxonomic composition), it would appear reasonable to expect that higher size variation among individuals would confer more among-individual diet variation. In particular, we expected higher size variance to increase between-individual diet variation (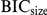 and 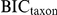), population niche width (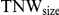 and 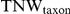), and the degree of diet variation (lower 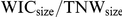, lower 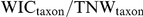 and higher E). Contrary to our expectations, stickleback size variance had no detectable effect on stickleback diet diversity in our experiment (Fig. 4). We observed no differences between treatments in size-based (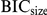; mean HV: 0.19; mean LV: 0.17; 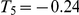, 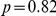) or taxon-based (E; mean HV: 0.80; mean LV: 0.81; 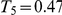, 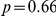) measures of diet variation, or any other diet index (all 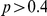; see Table 2 for detailed statistical results). In only one block did the HV enclosure have clearly higher diet variation (Fig. 4A), and this was driven largely by two individuals in the HV enclosure that consumed large numbers of the smallest prey type (copepod nauplii). This large value caused the residuals for 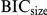, 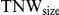 and 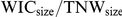 to be non-normally distributed, in spite of any attempted transformation. We repeated these analyses using non-parametric, two-sample Wilcoxon rank sum tests, with results remaining non-significant (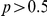).

**Table 2:** Treatment effects on population-level stickleback variables.

		mean HV	mean LV	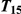	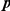
	Total Fish Biomass	35.8	34.12	−0.47	0.66
Morphology					
	Mean SL	**49.7**	**52.0**	**3.67**	**0.015**
	Variance in SL	**88.6**	**15.2**	**−6.49**	**0.001**
	Mean GRN	−0.05	0.10	0.92	0.40
	Variance in GRN	*1.84*	*2.64*	*2.07*	*0.093*
	Mean GRL	0.00	0.01	0.58	0.59
	Variance in GRL	0.04	0.03	−1.43	0.21
	Mean BD	−0.07	−0.06	0.29	0.79
	Variance in BD	0.19	0.18	−0.34	0.75
Diet					
	Mean Prey Size	1.56	1.61	0.81	0.45
	Slope (Diet vs. SL)	0.02	0.03	0.93	0.39
	Mean SC mass	2.31	2.24	−0.35	0.74
	E	0.80	0.81	0.47	0.66
	TNW	2.55	2.50	−0.77	0.48
	WIC	1.10	1.02	−1.09	0.33
	BIC	1.48	1.48	0.00	1.00
	WIC/TNW	0.44	0.41	−0.71	0.51
	BIC 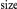 *	0.19	0.17	−0.24	0.82
	WIC 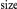	0.23	0.22	−0.38	0.72
	TNW 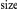	0.43	0.40	−0.50	0.64
	WIC/TNW 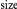 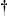	0.60	0.56	−0.42	0.69

Results of linear mixed-effects modeling of stickleback population-level response variables as a function of variance treatment and block effects (not shown). Significant or marginally significant effects are indicated in boldface (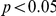) or italics (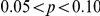). Variables marked with an asterisk (*) or dagger (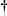) were log-transformed or exponentiated, respectively, to improve normality. SL = standard length, GRN = gill raker number, GRL = gill raker length, BD = body depth, SC = stomach content. Refer to text for definitions of diet indices.

### Post-hoc analyses

We anticipated that size variance would translate into diet variation, which might then have effects on lower trophic levels. Because size variance ultimately had no effect on diet variation, the expected chain of causation breaks down, explaining why size variance did not translate to effects on benthic or pelagic prey. Nevertheless, enclosures did exhibit variation in the degree of diet variation (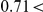
E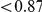) independent of our HV and LV treatments. We therefore tested for relationships between diet variation (E) and the four community variables under detectable top-down control in this experiment (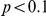; contrast between enclosures with and without fish): benthic invertebrate density, richness, evenness and composition (RDA axis 1). We included the spatial block effect but left size variance treatment out of the model. We also tested whether diet variation was related to total niche width (TNW), as is predicted under some models of ecological release, and has been demonstrated in stickleback and other taxa [46].

**Figure 5:**
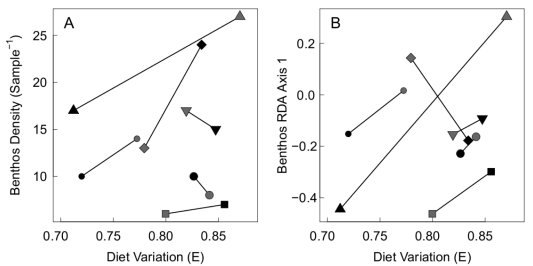
Post-hoc analysis of relationships between diet variation and the prey community in the enclosures. Mean pairwise diet dissimilarity (E) is compared to a) benthic invertebrate density and b) benthic invertebrate composition, measured as the first RDA axis. Symbols correspond to spatial blocks; black symbols indicate high size variance treatment, while grey symbols indicate low size variance.

These analyses revealed an intriguing, marginally significant relationship between diet variation and benthic invertebrate density (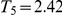, 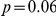; Fig. 5A). In any given block, the enclosure with greater diet variation tended to have higher benthos density, regardless of size variance treatment. There was also a non-significant trend for enclosures with greater diet variation to have invertebrate taxonomic composition more similar to fishless enclosures (RDA axis 1; 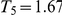, 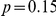; Fig. 5B), while there were no apparent relationships between E and benthos richness (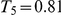, 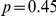) or evenness (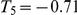, 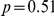). We also detected a positive relationship between E and TNW (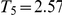, 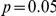), consistent with previous findings that more generalized populations exhibit greater among-individual diet variation [46]. We emphasize that although these analyses use diet variation as a predictor in place of size variance, diet variation itself was not manipulated in this experiment. We cannot therefore determine the direction of causation or exclude confounding factors, but can use these suggestive patterns as a guide for future research.

## Discussion

Our study fills a gap in the experimental literature by manipulating the level of variance in an ecologically important quantitative trait (body size) within a single population, and testing for effects on ecological interactions. We confirmed previous findings that body size is related to diet within age cohorts in stickleback population [30], [31]. For example, larger fish were more likely to consume certain larger prey types such as amphipods and larval mayflies, while smaller fish were more likely to consume smaller prey such as chydorids (logistic regression of prey presence/absence in stomach vs. SL of wild-caught fish; 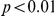). Therefore, we predicted that size variance would affect population niche width and among-individual diet variation. We also demonstrated top-down control by stickleback of their benthic invertebrate prey, suggesting that effects of size variance could in principle be detected.

The most important and surprising result of our study is that a 6-fold change in size variance in stickleback had no detectable effects on stickleback diet variation or niche width, let alone on the density or composition of lower trophic levels. This compelling lack of a variance effect runs counter to our expectations based on both theory and prior empirical results. It is therefore important that we carefully consider a range of factors that might (1) obscure an effect that actually exists, or (2) explain a genuine lack of relationship between trait variance and the ecological variables investigated here.

### Constraints on detecting a real community effect of intraspecific size variation

The absence of effects of size variance is fairly convincing, with no hint of a trend for almost all variables examined (Figs. 2 and 4). Nevertheless, we must consider the possibility that our experimental design had insufficient power to detect a real difference between high- and low-size variance treatments. We were able to detect strong effects of fish presence on several response variables, so if size variance had comparably large effects we were likely to have detected them. However, we may have had insufficient power to detect smaller, but still ecologically relevant, effects of fish size variance.

A second possibility is that intraspecific size variation typically does affect lower trophic levels, but that our experimental design did not effectively induce this effect. The strength of top-down control can vary over time [36], and our experiment tested for effects over only a three-week duration. A longer experiment might have induced a stronger effect, but two- or three-week enclosure experiments clearly allow detection of fish presence or density effects on top-down control (Fig. 2; [37]). The short duration of our study also has the advantage of minimizing the effects of mortality, growth or phenotypic plasticity that could alter our treatments over time. Spatial scale can also affect the magnitude and even direction of ecological experiments [47]. The enclosures we used were large enough to contain a realistic prey community, but small enough to ensure that all individual stickleback had equal access to all prey [33], [37].

The stickleback density used in this experiment results in natural levels of intraspecific competition, leading to prey densities and fish growth rates similar to those seen in the lake [37]. Elevated intraspecific competition at higher densities might have induced an effect of size variance on the prey community, as denser stickleback populations can further deplete prey and strengthen morphology-diet correlations in these enclosures [37]. Future experiments manipulating both population density and trait variance should help to determine whether variance has a stronger effect at higher densities. Any effect of size variance may also be weakened because of our use of semi-porous enclosure material that reduces but does not eliminate movement of prey into and out of the enclosures. High rates of diffusion through the enclosure netting may partly explain the lack of top-down control on zooplankton in this experiment, though at higher densities stickleback can deplete zooplankton abundance in these enclosures [37].

### Why might intraspecific size variation not have a community effect?

Next, we consider the possibility that intraspecific size variance in a predator really has a negligible effect on lower trophic levels. This might be the case if existing models adopt assumptions that do not apply to stickleback and their invertebrate prey. A central assumption of many ecological models of intraspecific trait variance is that individuals' traits map in a 1∶1 manner onto their resource use (e.g. [9], [11]). In contrast, we found a significant but only moderate correlation (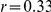; weighted 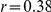) between the standard length and mean prey size of individual fish. Notably, the strength of this correlation is comparable to many other examples of intraspecific relationships between functional traits and resource use. For example, in a different stickleback population, foraging efficiency on pelagic prey is correlated with gill raker number and length, while efficiency on benthic prey is correlated with mouth width and SL (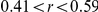; [32]). Beak size of the medium ground finch (*Geospiza fortis*) is correlated with use of large seeds (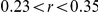; [48]), while in the brown anole (*Anolis sagrei*), hindlimb length is positively correlated with endurance running (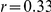), but negatively correlated with efficiency of running on narrow perches (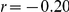; [49]). The relationship between SL and prey size in our study population is therefore comparable to many well-studied ecomorphological traits, but clearly less than 1∶1.

This relatively weak correlation between predator phenotype and diet may explain why phenotypic variance did not affect either diet variation or the prey community. While fish size was correlated with prey size, the range of mean prey sizes consumed by stickleback of intermediate size (e.g. the fish used in our LV treatment) spanned the entire range of mean prey sizes (Fig. 3A). Thus, while the size of a fish may predispose it to consume certain prey types, with a few exceptions any adult fish is capable of consuming prey of any size. Individuals may alter their prey choice in different competitive environments [37], [38], leading to similar population-level resource use (although this would predict a change in the slope of the relationship between mean prey size and SL, which we did not observe). These factors explain why, despite a correlation between size and diet, a large difference in size variance might have no detectable effect on total niche width or diet variation when this correlation is relatively weak.

Another possibility is that we chose to manipulate the wrong trait, as morphology more directly related to foraging might have a stronger effect on diet than size. Gill raker length and number, jaw structure, fin morphology and body shape influence how fish approach, strike at, and handle various prey types [28], [30], [32], and may generally be more accurate predictors of diet. Unfortunately, most of these traits can only be measured by invasive handling or dissection, so their variance cannot readily be manipulated. This is not a major limitation, however, because fish size was almost as good a predictor of diet composition as gill raker traits, and a better predictor than size-adjusted body depth. Therefore, we expect that variance in any single trophic morphological trait would have similarly weak effects. Ultimately, the most relevant traits are the ones that directly determine interaction strengths between species (preferences for particular prey types or microhabitats, attack rates and handling times), which may exhibit substantial variation not directly attributable to morphology.

Models might be wrong about the ecological effects of morphological variance (due to its weak correlation with diet), but still be correct that variance in resource use has considerable ecological effects. Our results illustrate this point: although size variance had no effect on benthic prey, stickleback diet variation did show intriguing relationships with benthos density and composition. Within a given spatial block, the enclosure with greater among-individual diet variation (E) tended to have benthos density and composition more similar to those in fishless enclosures. These results were statistically marginal and correlative in nature, meaning we cannot confidently infer causation. However, if the patterns are real, and if diet variation influences the benthic community rather than vice-versa, diet variation may reduce the strength of top-down control of stickleback on their prey. This would be consistent with theory suggesting that predators with nonlinear (i.e. type II) functional responses have lower overall consumption rates when there is among-individual variation in attack rates [4], [12].

The disconnect between trait variance and diet variation has a number of important implications. First, existing models of the ecological impact of intraspecific genetic or phenotypic variation should either incorporate noise into the mapping of morphology to resource use, or focus on variation in resource use traits themselves (which may be much more difficult to measure). Second, empiricists should find ways to manipulate ecologically relevant variance if we are to effectively study the ecological impacts of intraspecific variation. Finally, the insights presented here help to explain a classic conflict between theory and data. Van Valen's niche variation hypothesis predicted that populations experiencing ecological release would become more generalized via increased resource use variation among individuals [50]. Originally, this hypothesis was interpreted to mean that more generalist populations should show more variation in morphological traits. Many studies failed to find this correlation, but direct studies of diet variation have indicated that generalist populations do tend to show greater among-individual variation in diet [46]. Our results suggest that this conflict occurs because morphological variance is often a poor proxy for variation in resource use.

### Conclusion

Our intuition, codified in numerous theoretical models, is that morphological variation among individuals in a population can be used as a surrogate for niche variation. Even when morphology is indeed correlated with individual's resource use, this intuition may be wrong. Our analyses show that (1) fish size is correlated with prey size, and (2) stickleback suppress the density and alter the composition of their benthic invertebrate prey. Nevertheless, we found no effect of size variance on any measure of diet variation in experimental stickleback populations, or on the abundance or composition of their prey. However, analyses using diet variation in place of size variance hinted at effects on the prey community consistent with weaker top-down control. Thus, despite our largely negative results, intraspecific variation may still have profound effects on population, food web and ecosystem dynamics. The challenge ahead is to develop experimental approaches that explicitly test for such effects of intraspecific variation in resource use on ecological processes.
